# Supplementary material for: Urinary cortisol and cardiovascular events in women vs. men: The multi-ethnic study of atherosclerosis
Source: Am Heart J Plus. 2023 Nov 4;36:100344. doi: 10.1016/j.ahjo.2023.100344 (PMC10655947; doi:10.1016/j.ahjo.2023.100344)
Supplement: Supplementary file 1 [file mmc1.docx]

**SUPPLEMENTAL APPENDIX**

**SUPPLEMENT 1** 

**Description of formal second-order moderation testing.** Second order moderation analysis was performed with gender and central obesity as moderators for the associations between cortisol and cardiovascular event-free survival in the adjusted Cox models. Significant results were found across various levels of moderation: the three way term cortisol*gender*WHR was significant with HR 1.63 (1.04-2.56) and p-value 0.03, WHR moderation on cortisol was significant with HR 0.66 (0.53-0.83) and p-value 0.0004, WHR and gender moderation was significant with HR 0.14 (0.02-0.99) and p-value 0.04, and only the moderation between gender and cortisol was not significant with HR 1.09 (0.75-1.44) and p-value 0.80. Women were the referent level for gender.

Second order moderation analysis was also performed with gender and central obesity as moderators for the associations between cortisol and change in coronary artery calcium (CAC) in the adjusted Tobit regression models, with no significant results across various levels of moderation. The three-way annualized relative change term was 0.59 (0.32-1.08) and p-value 0.09, WHR moderation on cortisol was 1.20 (0.86-01.69) and p-value 0.280, WHR moderation on gender was 9.3 (0.70-124.46) and p-value 0.09, and the moderation between gender and cortisol was 0.83 (0.55-1.26) and p-value 0.37. Women were the referent level for gender.

A final second order moderation analysis was performed with race and central obesity as moderators for the associations between cortisol and cardiovascular event-free survival in the adjusted Cox models. There were no significant results across levels of moderation, with the three way cortisol*WHR*raceHispanic and cortisol*WHR*raceBlack having HR 0.88 (0.49-1.56), p-value 0.65, and HR 1.07 (0.56-2.01), p-value 0.84, respectively. 

**SUPPLEMENT 2**

**Table 3 Moderation hazard ratios.** Moderation hazard ratios can be interpreted as modifying HRs by the factor of the hazard ratio. For example, in women the median WHR was 0.93, and the HR between cortisol and new onset cardiovascular events was 0.95. Accordingly, in a group of women with WHR of 1.03 (an increase of 0.1 in WHR, or a change in 1 from the modeling perspective since WHR had been multiplied by 10 and centered), the HR between cortisol and new onset cardiovascular events would be expected to change by a factor of 0.67 to become 0.64 (from 0.67*0.95).

**SUPPLEMENT 3**

**Table 5 Annualized relative change**. Annualized relative change values from the Tobit regression models can be interpreted as that, for each doubling in cortisol, the ln(change in CAC score + 1) would change by a factor of the relative change each year. For an example of the magnitude of this effect, in women the interquartile range for cortisol was 9.5-24.3. This difference roughly corresponds with a doubling of cortisol, meaning that women at the highest quartile would be expected to have increased ln(change in CAC+1) by a factor of 1.60 each year compared to women in the lowest quartile. The median change in CAC in women in the lowest quartile of cortisol in this study was 0.6 per year. By putting this value into the regression equation, we find that in a one-year period women at the highest quartile of cortisol would be expected to have a median change in CAC of about 1.12 compared to 0.6 Hounsfield units in women at the lowest quartile of cortisol

| S1: New Onset Cardiovascular events and Urinary Cortisol Moderated by Waist-Hip Ratio, with Diabetes as a Covariate |  |  |  |
| --- | --- | --- | --- |
|  | Full cohort (n=918) HR (95% CI) | Women (n=491) HR (95% CI) | Men (n=427) HR (95% CI) |
| Cortisol, log2 ng / creatinine mg / dl | 1.04 (0.89-1.22) P=0.63 | 0.90 (0.71-1.15) P=0.40 | 1.04 (0.81-1.33) P=0.78 |
| WHR | **2.65** **(1.09-6.35)** **P=0.03** | **4.36** **(1.58-12.06)** **P=0.004** | 0.78 (0.13-4.71) P=0.78 |
| Cortisol*WHR | **0.79** **(0.64-0.97)** **P=0.02** | **0.65** **(0.51-0.83)** **P<0.001** | 1.04 (0.69-1.55) P=0.86 |
| WHR = waist-hip ratio. Models adjusted for age, race, annual income, smoking, alcohol, physical activity, systolic blood pressure, BMI, low-density lipoprotein levels, and diabetes. |  |  |  |

| S2: Associations between New Onset Cardiovascular Events and Cortisol by Median WHR, with Diabetes as a Covariate |  |  |  |  |  |  |  |  |  |
| --- | --- | --- | --- | --- | --- | --- | --- | --- | --- |
|  | Full cohort (n=918) |  | P for interaction | Women (n=491), |  | P for interaction | Men (n=427) |  | P for interaction |
|  | WHR≤0.95, N=459, HR (95% CI) | WHR>0.95 N=459, HR (95% CI) |  | WHR≤0.93 N=246, HR (95% CI) | WHR>0.93 N=245, HR (95% CI) |  | WHR≤0.98 N=214, HR (95% CI) | WHR>0.98 N=213, HR (95% CI) |  |
| Cortisol, ln ng / creatinine mg / dl | 1.20 (0.94-1.52) P=0.14 | 0.89 (0.71-1.11) P=0.30 | P=0.17 | 1.34 (0.93-1.92) P=0.12 | 0.75 (0.54-1.05) P=0.09 | **P=0.05** | 1.40 (0.99-1.97) P=0.06 | 0.94 (0.66-1.33) P=0.71 | P=0.28 |
| All values reported are hazard ratios generated from Cox regression analyses adjusted for age, race, annual income, smoking, alcohol, physical activity, systolic blood pressure, BMI, low-density lipoprotein levels, and diabetes.  Cortisol was log base 2 transformed for interpretability and to meet model assumptions. |  |  |  |  |  |  |  |  |  |

| **S3: Annual Change in Coronary Artery Calcium and Urinary Cortisol,** **with Diabetes as a Covariate** |  |  |  |
| --- | --- | --- | --- |
|  | Full cohort (n=447) Relative change (95% CI) | Women (n=245) Relative change (95% CI) | Men (n=202) Relative change (95% CI) |
| Cortisol, log2 ng / creatinine mg / dl | **1.37** **(1.11-1.68)** **P=0.003** | **1.52** **(1.13-2.06)** **P=0.006** | 1.18 (0.90-1.56) P=0.21 |
| WHR | 2.390 (0.74-7.17) P=0.15 | 0.96 (0.21-4.31) P=0.95 | 6.16 (0.73-52.19) P=0.10 |
| Cortisol*WHR | 0.97 (0.74-1.27) P=0.83 | 1.19 (0.83-1.71) P=0.34 | 0.74 (0.46-1.18) P=0.20 |
| WHR = waist-hip ratio. Models include age, race, annual income, smoking, alcohol, physical activity, systolic blood pressure, BMI, low-density lipoprotein levels, and diabetes. WHR was multiplied by 10 for interpretability and centered around the mean to meet model assumptions. |  |  |  |

| **S4: New Onset Cardiovascular events and Urinary Cortisol with Moderation by Body Mass Index** |  |  |  |
| --- | --- | --- | --- |
|  | Full cohort (918) HR (95% CI) | Women (n=491) HR (95% CI) | Men (n=427) HR (95% CI) |
| Cortisol | 1.03 (0.87-1.21) P=0.74 | 1.02 (0.80-1.31) P=0.87 | 1.04 (0.83-1.31) P=0.72 |
| BMI | 1.01 (0.91-1.12) P=0.90 | 1.09 (0.95-1.26) P=0.22 | 0.87 (0.73-1.04) P=0.14 |
| Cortisol*BMI | 1.01 (0.98-1.03) P=0.64 | 0.99 (0.96-1.02) P=0.60 | 1.04 (0.99-1.08) P=0.05 |
| BMI = body mass index, which was centered around the mean BMI. All values reported are hazards ratios generated from Cox regression analyses adjusted for age, race, annual income, smoking, alcohol, physical activity, systolic blood pressure, BMI, and low-density lipoprotein levels. |  |  |  |

| **S5: New Onset Cardiovascular events and Urinary Cortisol with Moderation by Waist Circumference** |  |  |  |
| --- | --- | --- | --- |
|  | Full cohort (918) HR (95% CI) | Women (n=491) HR (95% CI) | Men (n=427) HR (95% CI) |
| Cortisol | 1.03 (0.88-1.21) P=0.71 | 1.02 (0.80-1.29) P=0.90 | 1.02 (0.81-1.29) P=0.86 |
| WC | 1.01 (0.97-1.05) P=0.68 | 1.05 (0.99-1.11) P=0.09 | 0.96 (0.90-1.03) P=0.28 |
| Cortisol*WC | 1.00 (0.99-1.01) P=0.83 | 0.99 (0.98-1.01) P=0.37 | 1.01 (1.0-1.02) P=0.13 |
| WC = waist circumference, which was centered around mean waist circumference All values reported are hazards ratios generated from Cox regression analyses adjusted for age, race, annual income, smoking, alcohol, physical activity, systolic blood pressure, BMI, and low-density lipoprotein levels. |  |  |  |
